# Activation of Markers of Inflammation, Coagulation and Fibrinolysis in Musculoskeletal Trauma

**DOI:** 10.1371/journal.pone.0107881

**Published:** 2014-11-03

**Authors:** Olav Reikerås, Pål Borgen

**Affiliations:** 1 Institute of Clinical Medicine, Rikshospitalet, Oslo University Hospital, Oslo, Norway; 2 Martina Hansens Hospital, Oslo, Norway; Universidad Pablo de Olavide, Centro Andaluz de Biología del Desarrollo-CSIC, Spain

## Abstract

**Background:**

Traumatic injury induces changes in mediators of inflammation and coagulation, but the pivotal roles of inflammation and coagulation has not been precisely clarified. Therefore we have studied markers of inflammation and coagulation after a standardized musculoskeletal trauma like total hip replacement surgery.

**Methods:**

We allocated 21 patients aged 50 to 84 years who underwent total hip replacement surgery. Releases of TNF-α, IL-1β, IL-6, IL-8 and IL-10 and protrombin fragment F1.2 and plasmin-antiplasmin complex (PAP) were examined during surgery and up 6 days postoperatively, and systemic releases were compared to pre-operative values. Surgery induced significant increments in serum levels of IL-6 at 6 hours and at 1 day after surgery and in levels of IL-8 at 6 hours after surgery. There were no significant changes in serum levels of TNF-α, IL-1β or IL-10. There were significant increments in blood levels of F1.2 and PAP up to 6 days postoperatively with highest levels at 6 hours after surgery. There were only week correlations between IL-6 and IL-8 and F1.2 and PAP.

**Conclusion:**

Major musculoskeletal surgery causes changes of the inflammatory, coagulatory and fibrinolytic cascades in stable patients, but with no correlations between inflammation and coagulation and fibrinolysis.

## Introduction

Traumatic activation of inflammation and coagulation is recognized as a physiologic reaction to initiate healing and to act as a barrier to injury propagation and infection [1]. It arises from the interplay between various mediators produced at the site of injury, but with serious traumatic events, it can lead to a generalized state of inflammation referred to as the systemic inflammatory response syndrome with the potential to cause local and remote organ injury [2]. Inflammation and coagulation are intricately related processes that may considerably affect each other. A large number of inflammatory mediators, including cytokines have been shown to affect the coagulation cascade at several levels in monocyte and liver cells and promote inflammation in endothelial cells with structural and functional changes [3]. However, it has become increasingly clear, that vice versa, components of the coagulation system are able to markedly modulate the inflammatory response [3], [4]. Coagulation factors as well as anticoagulant proteins may activate specific cell receptors on mononuclear or endothelial cells, which may affect cytokine production or inflammatory cell apoptosis.

The entire pathophysiology of posttraumatic organ dysfunction is not fully understood, and an improved understanding of the pivotal roles of inflammation and coagulation seems important for directing appropriate patient care after traumatic injury [5]. To get insight into the physiological interactions between inflammation and coagulation following injury, we have studied markers of inflammation and coagulation and fibrinolysis after a standardized musculoskeletal trauma in the form of total hip replacement (THR).

## Methods

The study was approved by the Regional Ethics Committee of South East Norway (105-08012d 1.2007.1798. 1306./2058, 17679) and was performed in accordance with the ethical standards of the Declaration of Helsinki. We included 13 women and 8 men aged 60 to 84 years after written informed consent to participate in the study (Table 1). They all underwent primary cemented total hip arthroplasty (THA) due to osteoarthritis under spinal anesthesia with 5 mg/mL bupivacaine (Marcain; AstraZeneca, Södertälje, Sweden) injected at the lumbar level. The operation was performed in the lateral position, using a standardized posterior approach. Closed postoperative drainage was used for 24 hours. All patients were mobilized on the first postoperative day.

**Table 1:** Patients' characteristics. Values are mean ± standard deviation and (ranges).

Age (years)	70.6±7.8	(60–84)
Height (cm)	170±8.2	(155–192)
Weight (kg)	78.6±11.8	(63–108)
BMI	26.8±3.6	(22–33)
ASA classification	1.9±0.4	(1–2)

Thromboprophylaxis with low-molecular-weight heparin (Fragmin; Pharmacia & Upjohn, Stockholm, Sweden) and infectious prophylaxis with cephfalothin (Keflin; Eli Lilly, Indianapolis, IN., USA) were used. Voluven and Ringer's acetate (Fresenius KABI, Bad Homburg, Germany) were used as plasma substitutes. Postoperative analgesia was administered according to a standard protocol consisting of paracetamol and codeine sulphate (Paralgin forte; Weifa AS, Oslo, Norway) and ketobemidon (Ketorax; Jenahexal Pharma, Jena, Germany).

Patients with allergy to dalteparin, bleeding disorders, renal failure, hepatic disease, active treatment for malignancy, on-going antithrombotic treatment, history of deep vein thrombosis or pulmonary embolus, and patients experiencing major operations, traumas, stroke, or cardiac infarction the last 3 months before surgery were excluded. Patients were advised to stop antiplatelet medication and high-dose aspirin 1 week before surgery.

Hemoglobin, hematocrit, white blood counts, platelet counts, c-reactive-protein, creatinin, and liver enzymes were analyzed the day before surgery.

Blood samples were obtained from a peripheral vein at the following time points: before induction of anesthesia (T1), after induction of anesthesia, but before surgery (T2), at the end of surgery (T3), at 6 hours after surgery (T4), at the day after surgery (T5) and at 6 days after surgery (T6). Blood samples was kept on ice until it was separated by centrifugation at 2500 g for 20 min at 18 degrees C and stored at −80 degrees C until assayed. Analyzes of tumor necrosis factor α (TNF-α), interleukin 10 (IL-10) (R & D systems, MS, USA), IL-1β, IL-6 and IL-8 (CLB, the Netherlands) were performed by ELISA according to the manufacturers instruction.

Prothrombin fragment F1.2 and plasmin/α2-antiplasmin (PAP) were measured by ELISA by the use of commercial kit (Enzygnost F1.2 micro; Dade Behring, Marburg, Germany) following manufacturer's instructions.

Statistical analyses were performed using SPSS II software Version 19 (IBM Inc. USA). Data are presented by mean and standard deviation. Time dependent changes were performed by analysis of variance (ANOVA). If significant differences were indicated, we used the LSD post hoc test. Correlations and regression analyses were carried out, and P≤0.05 was considered significant.

## Results

The postoperative course was uneventful in all patients up to 6 days after surgery when they left the hospital. Before surgery CRP was 3.2±4.4 mg/L, at 3 days after surgery it was significantly increased to 83±53 mg/L (p<0.001), and it was still significantly increased at 6 days after surgery (46±31 mg/L) (p<0.001).

Surgery induced significant increments in serum levels of IL-6 at 6 hours after surgery (p<0.001) and at 1 day after surgery (p<0.001) and in levels of IL-8 at 6 hours after surgery (p = 0.004) (Table 2). There were no significant changes in serum levels of TNF-α (0.947), IL-1β (p = 0.421) or IL-10 (0.989).

**Table 2:** Changes in TNF-α, IL-1β, IL-6, IL-8, F 1.2 and PAP (pg/mL).

	T1	T2	T3	T4	T5	T6	ANOVA
TNF-α	0.8±1.7	0.9+1.5	0.6±1.0	0.7±0.8	0.6±0.9	0.8±1.0	0.854
IL-1β	8.3±18	9.2+19	4.2±11	4.0±11	3.9±12	11±20	0.602
Il-6	8.0±12	7.3+9.4	15±27	39±34^a^	40±31^a^	21±27	<0.001
Il-8	8.9±4.3	9.1+4.4	9.9±4.7	19+23**^b^**	9.8+4.6	15±67	0.019
Il-10	20±51	22+53	14±37	17±40	16±37	15±35	0.992
F 1.2	172±67	212+83	524±164^a^	573±148^a^	278±164**^c^**	350±109^a^	<0.001
PAP	542±143	535+138	907±259^a^	1039±276^a^	559±112	842±113^a^	<0.001

Time points are before induction of anaesthesia (T1), after induction of anaesthesias, but before surgery (T2), at the end of surgery (T3), at 6 hours after surgery (T4), at the day after surgery (T5), and at 6 days after surgery (T6). Values are mean ± standard deviation.

a p<0.001, **^b^**p = 0.004, **^c^**p = 0.013 in relation to T1.

There were significant increments in blood levels of F1.2 and PAP up to 6 days postoperatively (p<0.001) with highest levels at 6 hours after surgery. There were week correlations between serum levels of IL-6 and F1.2 and PAP (r = 0.194 and 0.066, respectively) and IL-8 and F1.2 and PAP (r = 0.183 and 0.193, respectively). By analyses of regression we found that serum levels of IL-6, IL-8, F1.2 or PAP were not significantly associated with age, sex and body mass index (BMI) (p = 0.636, 0.131 and 0.811, respectively).

## Discussion

Severe trauma results in the release of mediators of inflammation and coagulation, and sustained alterations have been associated with systemic complications [6], [7]. But the magnitude and relevance of such alterations in trauma patients who are physiologically stable are not widely appreciated. An important aspect is the link between coagulation and inflammation [8]. In our study we defined the insult in terms of a standardized surgical procedure. We found significant inflammatory, coagulatory and fibrinolytic responses following a major musculoskeletal injury in otherwise stable patients. However, there were no correlations between the markers of inflammation on one hand and the markers of coagulation and fibrinolysis on the other hand.

The age of our patients ranged from 60 to 84 years, and both women and men were included. Differences in age and sex as well as in nutritional status may influence the inflammatory response. However, the operations were done electively, all patients were well nourished as indicated by BMI, and there were no correlations between age, gender and BMI. Furthermore, we found no associations between age, gender and BMI on one side and inflammatory markers on the other. Second, it may be questioned whether the inflammatory response was influenced by the anesthetic. We measured markers before and after anesthesia, but before surgery, and we could not find any significant changes as a result of anesthetic. But as there is a rather short time interval between anesthesia and surgery, we can not say with certainty that anesthesia do or do not have inflammatory effects. Third, we did not measure the biomarkers locally. An increased production of pro-inflammatory mediators at the site of tissue damage may contribute to systemic inflammation and trauma-mediated immunosuppression [9].

The proinflammatory cytokines TNF-α, IL-1β, IL-6 and IL-8 and the antiinflammatory IL-10 have been considered important markers of inflammatory activity [10]. Therefore we focused on these. However, chemotactic cytokines govern the migration of inflammatory leukocytes into damaged areas, and typical inflammatory chemokines like CCL2 and CCL3 are known to play a key-role in the posttraumatic immune response [11]. These cytokines were also analyzed in our study, but as there were no significant changes in the systemic levels of these, they were not reported. Also, investigations into the use of IL-1β and IL-10 as clinical markers of the inflammatory response to trauma have been equivocal [12]. TNF-α is produced by a variety of cells and acts to increase the permeability of endothelial cells and the expression of adhesion molecules. But binding to its shed soluble receptors may interfere with measurement of serum levels [13]. Our results discount the value of the measurement of systemic TNF-α, IL-1β and IL-10 concentrations as acute markers of trauma and surgery.

In contrast, a direct relationship has been confirmed between elevated levels of IL-6 and IL-8 and degree of injury [14]. In a study evaluating clinical outcome in children following blunt trauma, serum IL-8 level at admission was identified as a most important determinant of postinjury mortality [15]. The increases in IL-8 in our study were short-lived at 6 hour after surgery and reflect that IL-8 is a chemokine that attracts polymorphonuclear neutrophil cells and macrophages into the wound site. Typically, at 24 h the neutrophil population is at its maximum, and the activity of these neutrophils may play a critical role in recovery. It has also been noted that systemic levels of IL-6 increase immediately following trauma and that patients with the most severe injuries have the highest levels [16]. A similar increase in venous levels of IL-6 during reamed intramedullary nailing of the femur was observed by Giannoudis et al. [17], and it is well accepted that elective surgical procedures cause an acute rise in venous levels of IL-6 in proportion to the magnitude and duration of the procedure [18]. Our observations on the systemic release of IL-6 concur with these investigations [19]. We found that IL-6 levels were increased at 6 and 24 h after surgery. This is in agreement with a previous study in stable trauma patients [20]. On the other hand, sustained high levels of IL-6 have been associated with increased severity of tissue injury and have correlated with subsequent development of post injury complications [18], [21].

IL-6 is the principal regulator of most acute-phase protein genes and regulates local and systemic inflammatory responses, including the synthesis of hepatic acute-phase reactants like C-reactive protein [22], [23]. We found increases in CRP like in Il-6. It has been suggested that IL-6 might partly be responsible for inducing the coagulatory cascade [24], and a positive correlation between IL-6 and prothrombin F1.2 concentrations has been noted [20]. F1.2 and PAP are accepted as specific markers of activation of the coagulation and fibrinolytic systems [25], and the systemic levels of these markers indicate the magnitude of tissue injury [26], [27]. Our results demonstrate a perioperative induction of these markers. We assume that intramedullary pressure during instrumentation cause intravasation of medullary contents with high levels of procoagulant factors [28], [29]. The perioperative increases in F1.2 might also be caused by passage into the lung of platelets that aggregate around fat emboli, thus inducing a systemic coagulatory response [30].

The immediate elevations in F1.2 and PAP preceded the increases in IL-6. The profile of F1.2 and PAP was decreasing the first postoperative day and then increasing until the 6the postoperative day. We assume that an unbalanced consumption and replenishment of coagulant and fibrinolytic factors explain the decreases the first postoperative day, followed by a hypercoagulable state that was prolonged after cessation of the inflammatory state. These findings harmonize with others and indicate a continuing procoagulant state even beyond hospital discharge in several patients [31]. As there were no correlations, our findings do not support the idea of a direct interaction between the inflammatory and the coagulatory cascade system in stable patients undergoing a major musculoskeletal trauma.

## Conclusion

Our study in stable patients undergoing a major musculoskeletal trauma indicates inflammatory and coagulatory and fibrinolytic responses with highest levels during the first postoperative day. But the processes of inflammation on one hand and coagulation and fibrinolysis on the other hand do not seem to affect each other.
